# Essential Oil Composition, Antioxidant, Cytotoxic and Antiviral Activities of *Teucrium pseudochamaepitys* Growing Spontaneously in Tunisia

**DOI:** 10.3390/molecules201119707

**Published:** 2015-11-16

**Authors:** Saoussen Hammami, Habib Jmii, Ridha El Mokni, Abdelbaki Khmiri, Khaled Faidi, Hatem Dhaouadi, Mohamed Hédi El Aouni, Mahjoub Aouni, Rajesh K. Joshi

**Affiliations:** 1 Research Unit Applied Chemistry and Environment 13ES63, Faculty of Sciences of Monastir, Monastir University, 5000 Monastir, Tunisia; khmiriabd@yahoo.fr (A.K.); faidi_khaled@yahoo.fr (K.F.); hatem.dhaouadi.fsm@gmail.com (H.D.); 2 Laboratory of Transmissible Diseases and Biologically Active Substances LR99ES27, Faculty of Pharmacy of Monastir, Avenue Avicenne, 5000 Monastir, Tunisia; jmiihbib@yahoo.fr (H.J.); mahjoub.ouni@fphm.rnu.tn (M.A.); 3 Laboratory of Botany and Plant Ecology, Faculty of Sciences, University of Bizerta, Jarzouna, 7021 Bizerta, Tunisia; riridah@yahoo.fr (R.E.M.); maitre.elaouni@yahoo.fr (M.H.E.A.); 4 Department of Phytochemistry, Regional Medical Research Centre (Indian Council of Medical Research), Belgaum, Karnataka-590010, India; joshirk_natprod@yahoo.com

**Keywords:** *Teucrium pseudochamaepitys*, chemistry, antioxidant, cytotoxic, antiviral

## Abstract

The chemical composition, antioxidant, cytotoxic and antiviral activities of the essential oil obtained by hydrodistillation from the aerial parts of *Teucrium pseudochamaepitys* (*Lamiaceae*) collected from Zaghouan province of Tunisia are reported. The essential oil was analyzed by gas chromatography equipped with a flame ionization detector (GC-FID) and gas chromatography coupled with mass spectrometry (GC/MS). Thirty-one compounds were identified representing 88.6% of the total essential oil. Hexadecanoic acid was found to be the most abundant component (26.1%) followed by caryophyllene oxide (6.3%), myristicin (4.9%) and α-cubebene (3.9%). The antioxidant capacity of the oil was measured on the basis of the scavenging activity to the stable 2,2-diphenyl-1-picrylhydrazyl (DPPH). The IC_50_ value of the oil was evaluated as 0.77 mg·mL^−1^. In addition, the essential oil was found to possess moderate cytotoxic effects on the HEp-2 cell line (50% cytotoxic concentration (CC_50_) = 653.6 µg·mL^−1^). The potential antiviral effect was tested against Coxsackievirus B (CV-B), a significant human and mouse pathogen that causes pediatric central nervous system disease, commonly with acute syndromes. The reduction of viral infectivity by the essential oil was measured using a cytopathic (CPE) reduction assay.

## 1. Introduction

Since prehistoric times, medicinal plants have been used as herbal formulations in crude forms, like tinctures, teas, powders and poultices, for their growing interest as alternative therapies for the prevention or treatment of various diseases [1]. Essential oils from medicinal and aromatic plants are still considered as rich sources of a huge number of antimicrobial and antifungal components [2]. Many of them show a great potential as anticancer therapeutic agents [3]. Plants from the *Lamiaceae* family are extensively explored in folkloric medicine, cosmetics, culinary applications and for the commercial production of essential oils [1]. *Teucrium* genus belonging to the *Lamiaceae* family comprises about 300 species, of which 23 form a part of the Tunisian flora [4]; this includes mostly perennial, rarely annual or biennial plants. Herbaceous subshrubs or even shrubs, they often show high aromatics [5]. A wide number of these species are rich in strongly-bioactive phenolic compounds and are used in folkloric medicine, in the food industry and in pharmacies for their antimicrobial, antinociceptive, antioxidant, hypolipidemic, anti-inflammatory and hypoglycemic properties [6]. Due to the wide spectrum of their biological activities, several essential oils from *Teucrium* aromatic plants play an important role to treat various human diseases [7]. According to the literature, only a few studies have revealed the antiphytoviral activity of pure essential oils; the phytochemical and biological investigations of *Teucrium polium*, *Teucrium flavum*, *Teucrium montanum* and *Teucrium chamaedrys*, widespread in the Croatian flora, showed that they are able to reduce cucumber mosaic virus (CMV) infections due to the high content in sesquiterpene hydrocarbons [6]. *Teucrium pseudochamaepitys*, growing spontaneously in Tunisia, is a perennial plant with raised stems (20–35 cm), ligneous and ramified on the basis. Leaves are divides into 3–5 linear strips and divaricated, with bluish-white flowers, curved and glandular calyx [8]. To our knowledge, phytochemists and biologists have paid no attention to this taxon; nothing was reported on the chemical composition and biological effects of crude and volatile extracts of *Teucrium pseudochamaepitys* anywhere in the world.

As a part of our work on the characterization of aromatic and medicinal plants growing spontaneously in Tunisia, we are now reporting the first studies on the chemical composition, the antioxidant and antiproliferative effects of the essential oil from the aerial parts of *Teucrium pseudochamaepitys* collected in the mountains of the Zaghouan region, northeast of Tunisia. To evaluate its possible use as an alternative or complementary cancer treatment, the antiviral activity was tested on Coxsackie 4 (CV-B4), a pathogenic enterovirus causing a wide range of human diseases, such as type 1 diabetes [9], myocarditis [10] and CNS pathologies among new-born and infants [11], by determining the concentration thatinhibited virus plaque formation and virus-induced cytopathogenicity by 50% (IC_50_).

## 2. Results and Discussion

### 2.1. Chemical Composition

Hydrodistillation of *Teucrium pseudochamaepitys* aerial parts provided a white essential oil with a yield of 0.15% (*w*/*w*). Although there are no reports about the productivity of essential oil from *T. pseudochamaepitys*, previous studies have shown that *Teucrium* species are generally rich in essential oils, for example the yield of *T. flavum* growing in Tunisia was 0.1% (*w*/*w*), and the productivity of *T. ramosissimum* and *T. marum* was 0.14% (*w*/*w*) and 0.59 (*v*/*w*), respectively [12,13,14]. The analyses and identification pointed out by mass fragmentation and retention indexes revealed the presence of 31 compounds, representing 88.6% of the total oil. The relative percentages of the main identified constituents are indicated in Table 1. From these results, it is clear that *Teucrium pseudochamaepitys* essential oil was dominated by hexadecanoic acid (synonym: palmitic acid, 26.1%). Apiole, caryophyllene oxide, myristicin, E-β-damascenone, α-cubebene, β-caryophyllene and elemicin were detected in appreciable amounts (7.1%, 6.3%, 4.9%, 4.6%, 3.9%, 3.5% and 3.3%, respectively).The oil was found to be rich in long chain hydrocarbons (51%), followed by sesquiterpene hydrocarbons (27.8%), oxygenated sesquiterpenes (6.3%) and oxygenated monoterpenes (3.5%).

**Table 1:** Chemical composition of *Teucrium pseudochamaepitys* essential oil. RI, retention index.

Compound	RI	%	Identification
Trans-sabinene hydrate	1058	0.9	RI,MS
Borneol	1131	1.8	RI,MS
Terpin-4-ol	1148	0.3	RI,MS
α-Terpineol	1161	0.5	RI,MS
Thymol	1282	2.9	RI,MS
Carvacrol	1292	1.5	RI,MS
Durenol	1325	0.4	RI,MS
α-Cubebene	1373	3.9	RI,MS
E-β-Damascenone	1391	4.6	RI,MS
α-Copaene	1404	1.0	RI,MS
β-Bourbonene	1412	0.8	RI,MS
β-Caryophyllene	1450	3.5	RI,MS
β-Humulene	1473	0.5	RI,MS
α-Humulene	1489	1.7	RI,MS
Dehydro-Aromadendrene	1487	1.3	RI,MS
E-β-Ionone	1510	0.3	RI,MS
γ-Muurolene	1511	2.8	RI,MS
Germacrene D	1520	0.7	RI,MS
α-Selinene	1525	0.3	RI,MS
Myristicin	1539	4.9	RI,MS
δ-Cadinene	1556	2.1	RI,MS
Cadine-1,4-diene	1568	3.1	RI,MS
α-Cadinene	1580	1.2	RI,MS
Elemicin	1584	3.3	RI,MS
1-nor-Bourbonanone	1589	0.2	RI,MS
Pentyl salicylate	1609	0.4	RI,MS
Caryophyllene oxide	1636	6.3	RI,MS
Apiole	1717	7.1	RI,MS
Myristic acid	1833	1.1	RI,MS
Pentadecanol	1873	3.1	RI,MS
Hexadecanoic acid	2030	26.1	
Oxygenated monoterpenes		3.5	
Sesquiterpene hydrocarbons		27.8	
Oxygenated sesquiterpene		6.3	
Other		51	
Total identified		88.6	

Previous chemical investigation of *Teucrium flavum* subsp. *flavum* essential oil revealed three chemotypes: β-caryophyllene (32.5%), α-humulene (17.8%) and germacrene D (6%) [12]. However, these compounds were detected in *T. pseudochamaepitys* essential oil at low concentrations (3.5%, 0.5% and 0.7%, respectively). The most abundant constituent of *T. pseudochamaepitys* species (palmitic acid, 26.1%) was not even detected in *T. flavum* oil. Thus, a high chemical variation of both species within the *Teucrium* genus originating from Tunisia has been detected.

### 2.2. DPPH Radical Scavenging Activity

The ability of the essential oil constituents to donate hydrogen atoms was measured using the stable free radical DPPH test. In the DPPH assay, antioxidants are typically characterized by their IC_50_ value, the concentration necessary to reduce 50% of DPPH radicals. Table 2 shows the percentage of DPPH inhibition of both *Teucrium pseudochamaepitys* essential oil and quercetin (standard solution) at different concentrations. These results indicate that the capacity of reducing the stable free 2,2-diphenyl-1-picrylhydrazyl (DPPH) to the yellow diphenylpicrylhydrazine increases with the concentration. The essential oil exhibited a significant scavenging effect with an IC_50_ of 770 µg·mL^−1^ (the concentration inducing 50% inhibition). These results proved that *Teucrium pseudochamaepitys* essential oil possesses more significant properties than *Teucrium flavum* collected in Tunisia and *Teucrium polium* collected in Iran (IC_50_ = 1230 µg·mL^−1^ and 9200 µg·mL^−1^, respectively).

**Table 2:** DPPH radical scavenging activity of *Teucrium pseudochamaepitys* essential oil.

DPPH Inhibition					
**Concentration**	1 mg·mL^−1^	0.5 mg·mL^−1^	0.25 mg·mL^−1^	0.125 mg·mL^−1^	IC_50_
**Essential oil**	51.89 ± 0.00	44.93 ± 1.90	35.43 ± 1.24	19.64 ± 3.19	0.77 ± 0.05
**Quercetin**	96.42 ± 0.45	92.57 ± 0.17	90 ± 0.72	89.57 ± 0.16	0.069 ± 0.02

### 2.3. Cell Viability Test

**Figure 1:**
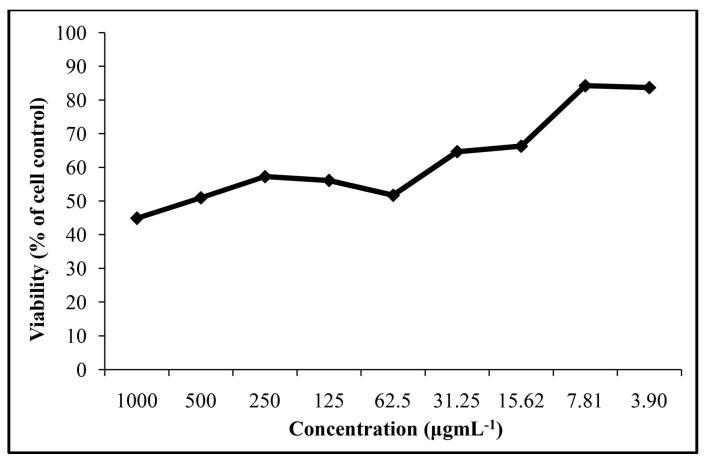
Cytotoxicity of *Teucrium* *pseudochamaepitys*.

To explore the potential use of *Teucrium pseudochamaepitys* essential oil as an antiviral agent, we first tested its possible *in vitro* acute and chronic toxicity. The toxicity in HEp-2 cells was evaluated using the MTT assay for essential oil samples. The assessment of cytotoxicity was performed for essential oil samples in the range of 1000 µg·mL^−1^–3.9 µg·mL^−1^. Dilution of the essential oil induced a dose-dependent inhibition of the cell cytotoxicity of the cell line (Figure 1). The half maximal toxic concentration 50% (50% cytotoxic concentration (CC_50_) of *Teucrium* oil on the cell line under examination was found to be 589.6 µg·mL^−1^. Thus, the essential oil was found to have moderate cytotoxicity against the HEp-2 cell line (100 µg·mL^−1^ ˂ CC_50_ ˂ 1000 µg·mL^−1^) [15]. However, the effective minimal concentration of *Teucrium pseudochamaepitys* essential oil should be below 7.81 µg·mL^−1^ for the antiviral assay.

### 2.4. Inhibition of CV-B Infectivity by Teucrium Essential Oil

**Figure 2:**
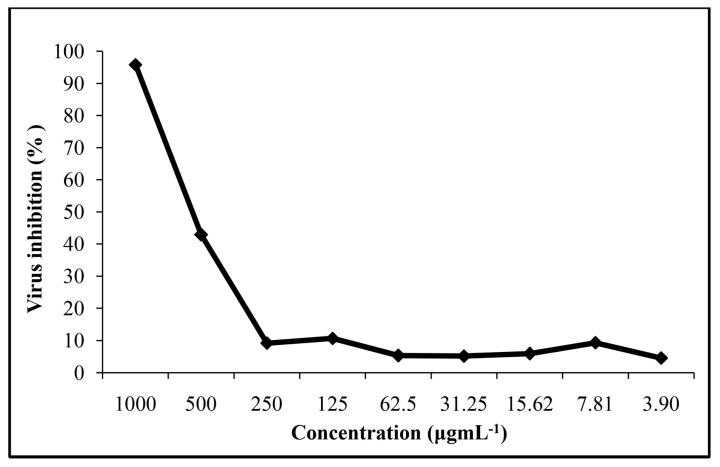
**Antiviral activity of *Teucrium pseudochamaepitys***.

The antiviral activity of *Teucrium pseudochamaepitys* essential oil was evaluated against Coxsackievirus B incubated at 37 °C with different concentrations of the volatile oil (Figure 2). The virus assay was based on the inhibition of virus-induced cytopathogenicity on HEp-2 cells. Results obtained from our screening demonstrated that the tested oil is ineffective against CV-B virus since the IC_50_ value was found to be 589.6 µg·mL^−1^ (Table 3) and the selectivity index was low, SI = 1.11 ˂ 3 [16,17]. In fact, antiviral activity is relevant when the extract tested has an IC_50_ value below 100 µg·mL^−1^ [16,17].

**Table 3:** Virucidal activity of *T. pseudochamaepitys* essential oil determined by the plaque reduction assay.

	CC_50_ (µg·mL^−1^)	IC_50_ (µg·mL^−1^)	SI	CC_80_ (µg·mL^−1^)	IC_80_ (µg·mL^−1^)
***Teucrium* essential oil**	653.6 ± 0.11	589.6 ± 0.46	1.11	2534.6 ± 0.08	852.3 ± 0.67

The 50% and 80% cytotoxic concentrations (CC_50_ and CC_80_), µg·mL^−1^, for *Teucrium* oil were calculated using linear regression analysis; the 50% and 80% inhibitory concentrations (IC_50_ and IC_80_), µg·mL^−1^, for *Teucrium* oil were calculated using linear regression analysis.

## 3. Experimental Section

### 3.1. Plant Material and Isolation of Essential Oil

The aerial parts of *T. pseudochamaepitys* were collected in the mountains of Zaghouan (northeast of Tunisia). Plant specimens were identified by one of the authors (R.E.M), botanist at the Laboratory of Botany and Plant Ecology, Faculty of Sciences of Bizerta, Jarzouna, Bizerta-Tunisia, where voucher specimens have been deposited. Air-dried material at ambient temperature (about 200 g) was subjected to hydrodistillation for 3 h in a Clevenger-type apparatus. The obtained oils were dried over anhydrous sodium sulfate.

### 3.2. Gas Chromatography

The GC analysis of the oil was carried out on a Varian 450 gas chromatograph equipped with FID, using a stationary phase CP Sil-8-CB (30 m × 0.25 mm i.d., 0.25-µm film thickness) column under the experimental conditions reported (Joshi, 2013a, 2013b) [16,17]. Nitrogen was a carrier gas at a 1.0 mL·min^−1^ flow rate. Temperature programming was 60–220 °C at 3 °C/min; for the injector and detector, temperatures were 230 and 250 °C, respectively. The injection volume was 1.0 µL of 1% solution diluted in *n*-hexane; the split ratio was 1:50.

### 3.3. Gas Chromatography-Mass Spectrometry

The GC-MS analysis of the oil was carried out on a Thermo Scientific Trace Ultra GC interfaced with a Thermo Scientific ITQ 1100 Mass Spectrometer fitted with TG-5 (30 m × 0.25 mm i.d., 0.25-µm film thicknesses) column. The oven temperature was programmed from 60–220 °C at 3 °C/min using helium as a carrier gas at 1.0 mL·min^−1^. The injector temperature was 230 °C; the injection volume 0.1 µL of 1% solution prepared in *n*-hexane; split ratio 1:50. MS were taken at 70 eV with a mass scan range of 40–450 amu. All of the experimental parameters were applied based on those reported earlier [18,19,20].

### 3.4. Identification of the Components

The identification of constituents was done on the basis of retention index (RI, determined with reference to homologous series of *n*-alkanes C_8_–C_25_, under identical experimental conditions), MS library search (NIST 08 Mass Spectra Library (Version 2.0 f) and WILEY’S Library of Mass spectra 9th Edition) and by comparison with MS literature data [21]. The relative amounts of individual components were calculated based on GC peak area (FID response) without using a correction factor.

### 3.5. Antioxidant Activity

DPPH radical scavenging assay: the 2,2’-diphenyl-1-picrylhydrazyl (DPPH) free radical assay was carried out to measure the free radical scavenging activity as reported previously [22]. A volume of 1.0 mL of each ethanol solution from Tunisian *T. pseudochamaepitys* prepared at different concentrations was mixed with an equal volume of ethanolic solution of DPPH (0.1 mM). The disappearance of the DPPH was measured after 30 min of incubation at room temperature. The inhibition percentage of the DPPH radical by the essential oil was calculated according to the formula of Yen and Duh [23].

% RSA = [(A_control_ − A_sample_)/A_control_] × 100 (1)

where A_control_ is the absorbance of the control sample (*t* = 0 h) and A_sample_ is the absorbance of a tested sample at the end of the reaction (*t* = 1 h).

The essential oil concentration providing 50% inhibition (IC_50_) was calculated from the graph plotting the percentage of radical scavenging activity (% RSA) against the Tunisian *T. pseudochamaepitys* essential oil concentration.

### 3.6. Viruses and Cell Line

The CV-B4 strain (kindly provided by Prof. J.W. Yoon, Julia M.C. Farlane, Diabetes Research Center, Calgary, AB, Canada) was multiplied in HEp-2 cells (Biowhittaker) in Eagle’s Minimal Essential Medium (MEM, Gibco BRL) supplemented with 10% FCS, 1% l-glutamine, 50 µg/mL streptomycin, 50 U/mL penicillin. Supernatants were collected three days post-infection (pi) and then clarified at 3000× *g* for 10 min. The virus titer was determined as the 50% tissue culture infectious dose on HEp-2 cells by the method of Reed and Muench (1938) and stored in aliquots at −80 °C until use.

### 3.7. Cell Seeding and Infection of Cell Cultures

The cytotoxic activity was tested against HEp-2 cells using the MTT assay [24]. HEp-2 cells were seeded at 5 × 10^4^ cells/well in 96-well plates and incubated at 37 °C/5% CO_2_ until 90% of confluency. Cells were washed 2 times with PBS before adding the compounds or the virus. In all of the experiments, cell control (cells that were not infected with the virus or treated with the compound) and virus control (cells that were infected only with the virus, but not treated with the compound in the antiviral assays) were taken into account.

### 3.8. Cytotoxicity Assay

The media of the 90% confluent HEp-2 cells were aspirated followed by the addition of 100 µL of each compound solution diluted in MEM 10% FCS (nine two-fold dilutions, ranging from 1000–3.9 µg·mL^−1^) and incubation for 72 h at 37 °C/5% CO_2_. Cell viability was assessed using 3-(4,5-dimethyl-2-thiazolyl)-2,5-diphenyl-(2*H*)tetrazolium bromide (MTT; Sigma, Saint Louis, MO, USA), which identifies living cells through the formation of formazan complexes, which was added to the washed cells with PBS. After 4 h of incubation at 37 °C/5% CO_2_, the reaction was stopped by adding DMSO. The absorbance of resulting formazan dye was measured on a spectrophotometer at wavelengths of 570 nm. Results are expressed as the percent viability compared to that for non-treated cells, for which viability was set to 100%. The cytotoxicity curve was then generated by plotting cell viability percentages against compound concentrations. Cell viability (%) was calculated for each concentration as Abs_treated_/Abs_cc_ × 100, where Abs_treated_ and Abs_cc_ are the absorbance readings for the wells with and without extract, respectively. The CC_50_ value was derived from the corresponding dose-response curves as the concentration of the oil that reduced cell viability by 50%. The maximum non-cytotoxic concentration is defined as the maximum concentration of the extract that leaves 100% viable cells.

### 3.9. Cytopathic Effect Inhibition Assay

The media of the 90% confluent HEp-2 cells were aspirated, and cells were inoculated with 50 µL of CV-B4 (100 TCID_50_) and simultaneously treated with 100 µL the compound solution diluted in DMEM/10% FCS (at nine two-fold dilutions, in the range of 1000–3.9 µg·mL^−1^) to each well. After incubation for three days at 37 °C/5% CO_2_, the results were quantified as described above. The virus inhibition percentages were measured using the following equation: T − Vc/Cc − Vc, where T is the optical density (OD) of compound-treated cells, Vc is the OD of virus control, Cc is the OD of cell control. The antiviral activity curve was then generated by plotting virus inhibition percentages against compound concentrations. The concentration that reduced 50% of CPE with respect to the virus control was estimated from the plots of the data and was defined as 50% inhibitory concentration (IC_50_). The selectivity index (SI) was calculated as the CC_50_/IC_50_ ratio [25].

## 4. Conclusions

In conclusion, the present study is the first to report the essential oil composition, antioxidant, cytotoxic and antiviral activities of *Teucrium pseudochamaepitys* growing spontaneously in Tunisia. The bioactivity evaluation of the essential oil demonstrates a more significant antioxidant activity than that of *T. flavum* and moderate cytotoxic effects on the HEp-2 cell line. Thus, further phytochemical investigations of *T. pseudochamaepitys* crude and volatile extracts to isolate the biologically-active compounds will be of great importance.
